# HGS-ETR1, a fully human TRAIL-receptor 1 monoclonal antibody, induces cell death in multiple tumour types *in vitro* and *in vivo*

**DOI:** 10.1038/sj.bjc.6602487

**Published:** 2005-04-20

**Authors:** L Pukac, P Kanakaraj, R Humphreys, R Alderson, M Bloom, C Sung, T Riccobene, R Johnson, M Fiscella, A Mahoney, J Carrell, E Boyd, X T Yao, L Zhang, L Zhong, A von Kerczek, L Shepard, T Vaughan, B Edwards, C Dobson, T Salcedo, V Albert

**Affiliations:** 1 Human Genome Sciences Inc., 9800 Medical Center Drive, Rockville, MD 20850, USA; 2 Cambridge Antibody Technology, Milstein Building, Granta Park, Cambridge CB1 6GH, UK

**Keywords:** TRAIL receptor 1, TRAIL, apoptosis, antibody, chemotherapeutic agents

## Abstract

Tumour necrosis factor-related apoptosis-inducing ligand (TRAIL) induces apoptosis in a variety of tumour cells through activation of TRAIL-R1 and TRAIL-R2 death signalling receptors. Here, we describe the characterisation and activity of HGS-ETR1, the first fully human, agonistic TRAIL-R1 mAb that is being developed as an antitumour therapeutic agent. HGS-ETR1 showed specific binding to TRAIL-R1 receptor. HGS-ETR1 reduced the viability of multiple types of tumour cells *in vitro*, and induced activation of caspase 8, Bid, caspase 9, caspase 3, and cleavage of PARP, indicating activation of TRAIL-R1 alone was sufficient to induce both extrinsic and intrinsic apoptotic pathways. Treatment of cell lines *in vitro* with HGS-ETR1 enhanced the cytotoxicity of chemotherapeutic agents (camptothecin, cisplatin, carboplatin, or 5-fluorouracil) even in tumour cell lines that were not sensitive to HGS-ETR1 alone. *In vivo* administration of HGS-ETR1 resulted in rapid tumour regression or repression of tumour growth in pre-established colon, non-small-cell lung, and renal tumours in xenograft models. Combination of HGS-ETR1 with chemotherapeutic agents (topotecan, 5-fluorouracil, and irinotecan) in three independent colon cancer xenograft models resulted in an enhanced antitumour efficacy compared to either agent alone. Pharmacokinetic studies in the mouse following intravenous injection showed that HGS-ETR1 serum concentrations were biphasic with a terminal half-life of 6.9–8.7 days and a steady-state volume of distribution of approximately 60 ml kg^−1^. Clearance was 3.6–5.7 ml^−1^ day^−1^ kg^−1^. These data suggest that HGS-ETR1 is a specific and potent antitumour agent with favourable pharmacokinetic characteristics and the potential to provide therapeutic benefit for a broad range of human malignancies.

Tumour necrosis factor-related apoptosis-inducing ligand (TRAIL), also known as Apo2L, is a member of the TNF ligand superfamily (Wiley *et al*, 1995; Pitti *et al*, 1996). It induces apoptosis in many cancer cell lines, with minimal to no effect on most normal cells (Wiley *et al*, 1995; Pitti *et al*, 1996; Kothny-Wilkes *et al*, 1998; Ashkenazi *et al*, 1999; Walczak *et al*, 1999; Lawrence *et al*, 2001; Evdokiou *et al*, 2002). TRAIL mediates apoptosis through two death receptors, TRAIL-receptor 1 (TRAIL-R1, DR4, TNFRSF10A) and TRAIL-receptor 2 (TRAIL-R2, DR5, TNFRSF10B). While additional receptor proteins that bind TRAIL exist (TRAIL-R3/DcR1, TRAIL-R4/DcR2, Osteoprotegerin/OPG), these receptors lack functional death domains and, as such, do not initiate apoptosis (LeBlanc and Ashkenazi, 2003; MacFarlane, 2003).

The TRAIL death receptor-induced apoptosis is mediated through activation of both extrinsic and intrinsic intracellular death signalling pathways. TRAIL binding to death receptors results in formation of a death-inducing signalling complex (DISC), consisting of death receptors, adaptor proteins, and procaspase 8, which leads to processing and activation of procaspase 8 by an autocatalytic mechanism (Sprick *et al*, 2000). Activated caspase 8 triggers the extrinsic apoptotic pathway by directly activating effectors such as caspase 3 and caspase 7, resulting in cleavage of downstream targets such as poly (ADP-ribose) polymerase (PARP). Caspase 8 can also initiate the intrinsic apoptotic pathway through the activation of Bid. Activated Bid induces oligomerisation of proapoptotic proteins Bax and Bak, resulting in the release of both cytochrome *c* and Smac/DIABLO from the mitochondria and subsequent activation of caspase 9 (Srivastava, 2001; Ozoren and El-Deiry, 2003). Both pathways lead to the activation of caspase 3 and eventual apoptotic cell death.

TRAIL induces apoptosis in various tumour cell types *in vitro* and *in vivo*. While TRAIL alone induces apoptosis in sensitive cancer cell lines, TRAIL activity can be enhanced upon coexposure with various chemotherapeutic agents (Gliniak and Le, 1999; Keane *et al*, 1999; Yamanaka *et al*, 2000; Bouralexis *et al*, 2001; Evdokiou *et al*, 2002; Shimoyama *et al*, 2002). Tumour cells that are resistant to TRAIL can be sensitised by combination treatment with various chemotherapeutic drugs (LeBlanc *et al*, 2002). *In vivo*, the antitumour efficacy of TRAIL in combination with chemotherapeutic agents, such as 5-fluorouracil and irinotecan, was greater than the activity of either agent alone (Gliniak and Le, 1999; Nagane *et al*, 2000; Naka *et al*, 2002; Ray and Almasan, 2003; Singh *et al*, 2003).

In addition to TRAIL, other methods of targeting TRAIL receptors have been proposed as cancer therapeutics. Agonistic TRAIL-R1 or TRAIL-R2 antibodies may have enhanced therapeutic potential due to a prolonged half-life *in vivo* compared to TRAIL ligand (Yagita *et al*, 2004). Proof of concept has been demonstrated with murine or rabbit monoclonal antibodies (mAbs) to human TRAIL-R1 or TRAIL-R2, which have antitumour activity *in vitro* and *in vivo* (Griffith *et al*, 1999; Muhlenbeck *et al*, 2000; Chuntharapai *et al*, 2001; Ichikawa *et al*, 2001). Mechanistically, these agonistic antibodies work by activation of TRAIL receptor-mediated apoptotic pathways in a manner similar to TRAIL, as a TRAIL-R1 antibody induced PARP cleavage in B-cell lymphoma 9D cells (Chuntharapai *et al*, 2001), and TRAIL-R2 antibodies induced activation of caspases and JNK/p38 kinase in tumour cells (Ichikawa *et al*, 2001; Ohtsuka *et al*, 2003). Enhanced apoptotic signalling and cell killing *in vitro* has been observed with TRAIL-R1 and TRAIL-R2 antibodies in combination with chemotherapeutic agents (Ohtsuka *et al*, 2003). A murine TRAIL-R2 monoclonal antibody demonstrated inhibition of tumour growth in a breast cancer xenograft model alone and in combination with chemotherapeutic agents and radiation (Buchsbaum *et al*, 2003). These results underscore the potential use of agonistic TRAIL-receptor antibodies for cancer treatment; however, use of fully human antibodies would be preferable for clinical applications to avoid the immunogenicity associated with mouse and rabbit antibodies (Berger *et al*, 2002).

Here, we describe the development and characterisation of HGS-ETR1 (Mapatumumab), a fully human agonistic monoclonal antibody with high affinity and specificity for TRAIL-R1. HGS-ETR1 induced cell death in tumour cell lines and this killing was mediated through the activation of both extrinsic and intrinsic death signalling pathways. HGS-ETR1 was shown to have a long half-life *in vivo* and suppressed the growth of colon, lung, and renal tumours in xenograft models in athymic mice. HGS-ETR1 also enhanced the antitumour efficacy of chemotherapeutic drugs. These results show that HGS-ETR1 is a potent antitumour agent either used alone or in combination with other therapeutic drugs.

## MATERIALS AND METHODS

### Cell culture and reagents

Tumour cell lines Colo205, HCT116, H460, H2122, ST486, SW480, RL95-2, SU.86.86, ES2, A498, WM793, and SNU398 (Park *et al*, 1995) were obtained from American Type Culture Collection (ATCC, Rockville, MD, USA). JURL-MK1 tumour cell line was obtained from Deutsche Sammlung von Mikroorganismen und Zellkulturen GmbH (DSMZ, German Collection of Microorganisms and Cell Cultures, Mascheroder, Germany). TTn tumour cell line was obtained from the Japanese Collection of Research Bioresources (JCRB Cell Bank, Tokyo, Japan). Tumour cell lines were cultured and cell assays performed in media recommended by supplier containing at least 10% serum. Chemotherapeutics sources were: topotecan, Calbiochem (San Diego, CA, USA); cisplatin, Bristol Myers-Squibb (Princeton, NJ, USA); irinotecan, Pharmacia & Upjohn (Kalamazoo, MI, USA); 5-fluorouracil (5FU), American Pharmaceutical Partners, Inc. (Schaumburg, IL, USA); and carboplatin and camptothecin, Sigma (St Louis, MO, USA).

### Selection and generation of a TRAIL-R1 agonist monoclonal antibody

Phage display was used to isolate anti-TRAIL-R1 antibodies. A large nonimmunised human scFv (single chain variable fragment) phage display library was used for selection as described previously (Marks *et al*, 1991). Human soluble extracellular domain (ECD) TRAIL-R1-flag fusion protein at 10 *μ*g ml^−1^ in PBS was immobilised onto immunotubes (Nunc, Rochester, NY, USA) overnight at 4°C. Three or four rounds of panning selections were performed using the scFv human antibody phage library with approximately 1.4 × 10^10^ individual recombinants (Vaughan *et al*, 1996). A deselection round was performed on an irrelevant fusion protein to remove any nonspecific binders. From this process, a panel of 1500 clones were selected and screened by ELISA for binding to TRAIL-R1. Screening of clones by ELISA has been described previously (Jackson *et al*, 1992). A total of 250 scFvs were identified that bound to TRAIL-R1, but did not bind to an irrelevant fusion protein. DNA sequence analysis of this panel identified 102 unique scFvs. Candidate scFvs were further selected from this panel based on agonistic activity in viability assays of SW480 and HeLa cells. The scFvs with the highest activity, including HGS-ETR1, were converted to IgG1, expressed in an NSO mouse myeloma cell line, secreted into culture media, and purified by a series of standard chromatography steps.

### ELISAs

To determine direct binding of fully human IgG1 HGS-ETR1 to TRAIL-R1, TRAIL-receptor extracellular domain fusion proteins (TRAIL-R1, -R2, -R3, -R4) were immobilised on 96-well plates, and various concentrations of HGS-ETR1 (0.9–66.7 nM) were added. After incubation for 2 h at room temperature, the wells were washed and bound HGS-ETR1 antibody was detected using peroxidase-conjugated goat anti-human IgG Fab and tetramethylbenzidine (TMB) horseradish peroxidase (HRP) substrate solution (KPL, Gaithersburg, MD, USA) according to the manufacturer's instructions.

### Flow cytometry

Adherent cell lines were detached from flasks using EnzFree buffer (Quality Biological, Gaithersburg, MD, USA), washed with FACS buffer (PBS with 0.1% NaN_3_ and 0.1% BSA) and resuspended at 10^6^ cells per 100 *μ*l. Cell surface expression of TRAIL receptors was determined using 10 *μ*g ml^−1^ (final concentration) of HGS-ETR1 or commercial mouse anti-TRAIL-R1 antibodies (eBiosciences, San Diego, CA, USA). Matched isotype control human IgG (IC mAb) or commercial mouse IgG (eBiosciences) monoclonal antibodies were used as negative controls. Cells were incubated with antibodies for 20 min at room temperature, washed, resuspended in 200 *μ*l FACS buffer containing 0.5 *μ*g ml^−1^ propidium iodide and analysed on a FACScan (Becton Dickinson, Franklin Lakes, NJ, USA). To determine specificity of HGS-ETR1 binding, FACS analysis was performed in the presence of 5 *μ*g ml^−1^ of soluble TRAIL receptor-Fc fusion proteins (R&D Systems, Minneapolis, MN, USA).

### Viability assays

Cell viability was determined using Cell Titer-Glo (Promega, Madison, WI, USA). Cells were plated in 96-well white polystyrene opaque plates (Costar/Corning, Acton, MA, USA) and incubated overnight at 37°C. Indicated concentrations of HGS-ETR1 antibody or controls were added. In combination studies, chemotherapeutic agents were also added at the indicated concentrations with either control antibody or HGS-ETR1. After 48 h incubation, cell viability was measured according to the manufacturer's instructions. In brief, 100 *μ*l of assay reagent was added to cells at room temperature, mixed and incubated for approximately 30 min. Luminescent signal was read using a Northstar luminescent plate reader. All treatments were performed in triplicate. The average and standard deviation were determined and data plotted using Prism software (GraphPad Software, San Diego, CA, USA).

### Fluorescent caspase assays

Cells were plated in black-walled 96-well plates (Costar) at 1 × 10^4^ cells well^−1^ and cultured overnight. Cells were treated with HGS-ETR1 or controls for the indicated times at 37°C and caspase activity was measured using a tagged caspase substrate (rhodamine-labeled DEVD peptide). The assay was performed according to the manufacturer's instructions (Apo-ONE Homogeneous Caspase 3/7 Assay, Promega) and the release of the fluorogenic moiety by activated caspases was measured by reading plates at 405 nm using a fluorometric plate reader. Treatments were performed in triplicate and the average and standard deviation determined and plotted.

### Western blot analysis

Cells (2 × 10^6^) were plated overnight in a 150-mm cell culture plate and then treated with the indicated concentrations of HGS-ETR1 or control antibody for 4 h at 37°C in a cell culture incubator. Cells were then scraped in ice-cold PBS, centrifuged and cell pellets lysed with 1% NP40 lysis buffer (10 mM HEPES pH 7.5, 0.15 mM NaCl, 10% glycerol, protease inhibitors cocktail and 1 mM PMSF). The protein concentration of the lysates was determined by BCA method (Pierce, Rockford, IL, USA) and normalised with lysis buffer. The proteins were separated using Tris-glycine polyacrylamide SDS gel electrophoresis and transferred to nitrocellulose membranes (Invitrogen, San Diego, CA, USA). The filters were incubated with antibodies that recognise the pro and cleaved forms of the apoptotic proteins PARP (Pharmingen, San Diego, CA, USA), caspase 3, caspase 8, caspase 9 (Upstate Biotechnology Inc., Lake Placid, NY, USA), Bid (Cell Signaling, Beverly, MA, USA), and actin (Santa Cruz, Santa Cruz, CA, USA). The bands corresponding to specific proteins were detected by HRP-conjugated secondary antibodies and enhanced chemiluminescence (Amersham Corp., Piscataway, NJ, USA).

### *In vivo* tumour models

For pre-established xenograft tumour models (Colo205, HCT116, H2122, and A498), female Swiss athymic mice (Taconic, Germantown, NY, USA), 7–8 weeks of age and 20 g average body weight, were used. All experiments were performed in accordance with the current guidelines established by UKCCR and the Institutional Animal Care and Use Committee at Human Genome Sciences Inc. On day 0, 1 × 10^7^ tumour cells were injected subcutaneously (s.c.) in the lower right flank of the mouse (H2122, Colo205, A498, HCT116). Prior to any treatments, tumours were grown to ∼100 mm^3^ and 8–10 mice per group were used for each treatment group. In the single-agent models, HGS-ETR1 or isotype control antibody was administered to the animals intravenously (i.v.) via tail vein in a dose/weight-matched fashion on indicated days. Tumours were established as above for the Colo205 and HCT116 combination treatment models. In the Colo205 combination model, 5FU mini-pumps were inserted on day 7. Each 0.5 ml h^−1^ mini-osmotic pump (Alzet Osmotic Pumps, Durect Corporation, Cupertino, CA, USA) was filled with sterile 5FU solution (50 mg 5FU ml^−1^ water, pH 9.0) for a 1.25 mg kg^−1^ h^−1^ flow rate. Mice were anesthetised with inhaled isoflurane, skin was sterilised and the mini-osmotic pumps containing 5FU were inserted s.c. in a pocket formed dorsally under the skin of the mouse. Skin was closed with two 9 mm wound clips. Wound clips were removed after 14 days. Continuous infusion of 5FU was given from days 7–21. Isotype control antibody (10 mg kg^−1^) was administered i.v. on days 7, 10, 12, 14, 17, 19, 21, 24, 26, 28, and 31. HGS-ETR1 (10 mg kg^−1^) was administered i.v. on days 14, 17, 19, 21, 24, 26, 28, and 31 either alone or in animals treated with 5FU. One group of animals was untreated. In the HCT116 combination treatment model, HGS-ETR1 or isotype control antibody (10 mg kg^−1^) was administered i.v. on days 11, 17, and 24. Irinotecan was administered i.p. at a dose of 8 mg kg^−1^ on days 11, 15, 17, 21, 24, and 28. For the SW480 combination treatment *de novo* model, male Swiss athymic mice (Taconic), 6–8 weeks of age and 20–25 g average body weight, were used and 1 × 10^7^ SW480 cells were injected per site, six mice per group on day 0. On day 1, a loading dose of HGS-ETR1 (20 mg kg^−1^) was administered i.v. Topotecan was administered i.p. at a dose of 0.3 mg kg^−1^ on days 1–3. Subsequently, HGS-ETR1 (10 mg kg^−1^) and topotecan (0.3 mg kg^−1^) were administered i.p. on days 4, 7, 10, 13, and 16. One group of mice was injected with vehicle control. For all models, tumour size on two axes was measured with digital calipers at 3–4-day intervals. The values were transformed into tumour volumes using the following formula: tumour volume=0.5 × width^2^ × length. Unpaired Student *t*-tests were used to evaluate the significance of differential tumour growth. The difference was considered significant when *P* was <0.05.

### *In vivo* pharmacokinetics

Male BALB/c mice (8 weeks old, 21–25 g) were obtained from Ace Animals (Boyertown, PA, USA). Mice were injected i.v. via tail vein with 10 or 20 mg kg^−1^ HGS-ETR1. Four mice were injected for each time point. Mice were killed and blood was collected at 5 min, 3 h, 8 h, 1, 2, 3, 4, 7, and 10 days postinjection. Sera prepared from the blood samples were stored at −80°C until analysed by ELISA. For the ELISA assays, a Maxisorb plate (Nalge Nunc International, Rochester, NY, USA) was coated with a TRAIL-R1 fusion protein solution (2 *μ*g ml^−1^ in PBS), and blocked with a PBS solution containing 10% goat serum. The sera samples to be tested for HGS-ETR1 levels were diluted 2000-fold (0.05%) in a PBS solution containing 0.05% Tween-20. HGS-ETR1 standards and positive controls were made in a PBS solution containing control 0.05% BALB/c serum. Diluted samples, standards, and positive controls were incubated on the plate for 2 h at room temperature. The plate was washed six times using a PBS solution containing 0.1% Tween-20 and incubated for 2 h with a PBS solution of 10% goat sera containing HRP-labelled anti-human *λ*IgG antibody (1 : 80 000). The plate was washed once more prior to the addition of TMB HRP substrate. After a 15-min development time, the HRP activity was terminated using dilute sulphuric acid and the relative absorbance at 450 nm was measured using a Spectromax ELISA (Molecular Devices, Sunnyvale, CA, USA) plate reader. The concentration of the HGS-ETR1 in the sera samples was extrapolated from the HGS-ETR1 standard curve. The limit of quantitation of the assay is 5 ng ml^−1^ in 100% serum. To obtain pharmacokinetic parameters, serum concentration data were fitted with a two-compartment i.v. bolus model with 1/(predicted value)^2^ weighting using the software package WinNonlin (Pharsight Corp., Mountain View, CA, USA). Data from all mice in each dose group were fit together to obtain a pooled estimate of the PK parameters.

## RESULTS

### HGS-ETR1 binds specifically to human TRAIL-R1

**Figure 1:**
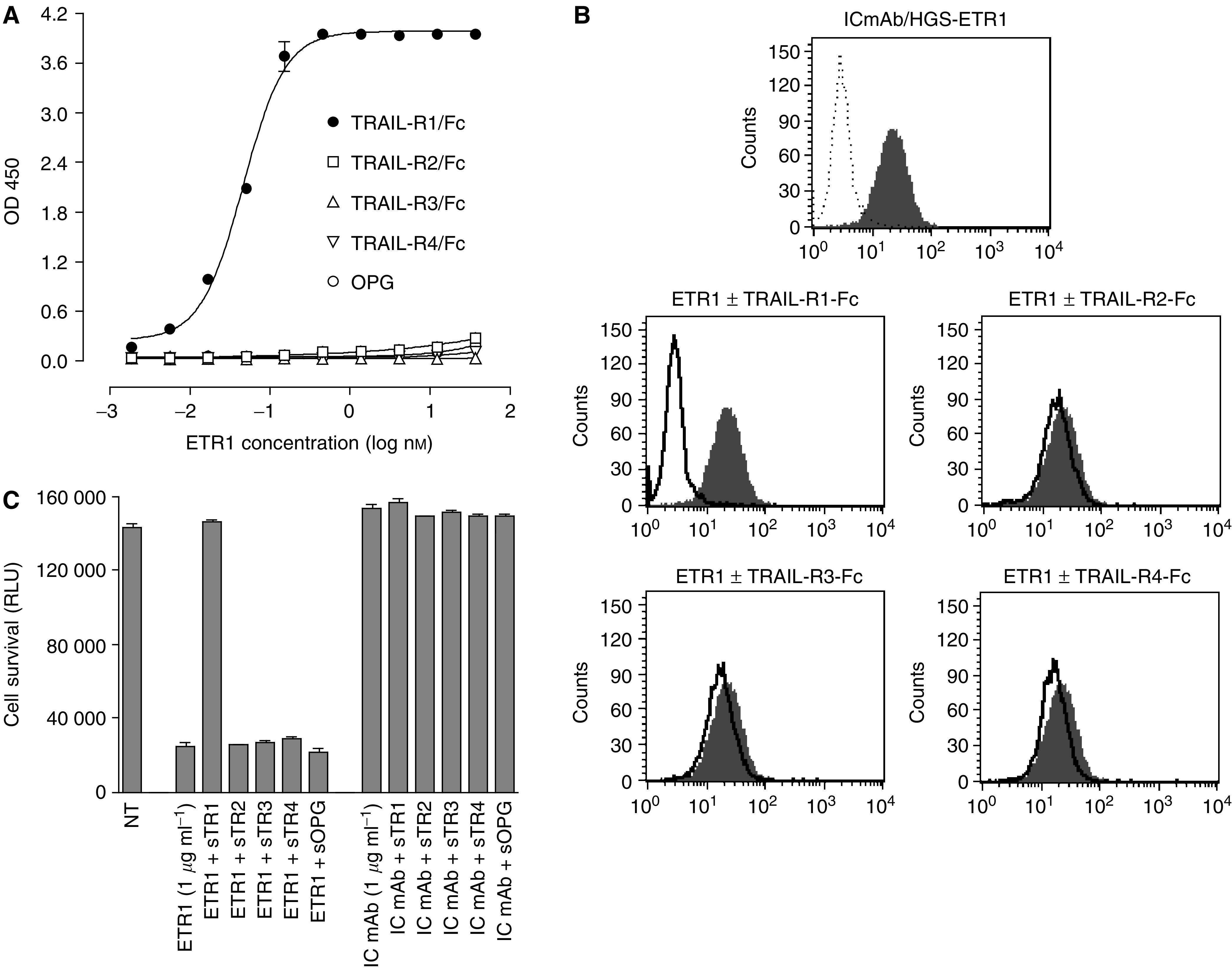
Characterization and specificity of HGS-ETR1 antibody. (**A**) Direct-binding ELISA data show the binding of indicated concentrations of HGS-ETR1 to the TRAIL-R1 extracellular domain Fc-protein (filled circles) or other TRAIL-R extracellular domain Fc-proteins (open symbols) immobilised on 96-well plates. Binding of HGS-ETR1 to TRAIL-receptors was detected using anti-human Fab-peroxidase conjugate. (**B**) Binding of HGS-ETR1 to SW480 cells analysed by flow cytometry. The top panel shows the binding of isotype control monoclonal antibody (IC mAb, dotted line) and HGS-ETR1 (shaded peak). The bottom panels show the binding of HGS-ETR1 alone (shaded peak) or binding of HGS-ETR1 in presence of 5 *μ*g ml^−1^ of the indicated soluble TRAIL-R Fc-protein (solid line). (**C**) For viability assays, SW480 cells were plated at 1 × 10^4^ cells well^−1^ in a 96-well plate and cultured overnight. A 1 *μ*g ml^−1^ concentration of HGS-ETR1 or isotype control monoclonal antibody (IC mAb) was added to the cells in the absence or presence of the indicated soluble TRAIL-receptor proteins (sTRAIL-R1-4, sOPG, 5 *μ*g ml^−1^) and cell viability, measured as relative light units (RLU), was determined after 48 h. Data is the mean±s.d. of triplicate samples.

TRAIL-R1-specific scFv antibodies were isolated from human phage display libraries based on their binding to a TRAIL-R1 extracellular domain fusion protein. A panel of 102 unique anti-TRAIL-R1 scFv antibodies was further screened for inhibition of TRAIL binding to TRAIL-R1 and for cytotoxic effects on HeLa cells (data not shown). Several top candidates were converted to IgG_1_ and further characterised for agonist activity on multiple tumour cell types and antitumour activity in xenograft models. HGS-ETR1 was selected as the lead candidate based on its potent *in vitro* and *in vivo* antitumour effects. Binding of the fully human IgG_1_ form of HGS-ETR1 to the extracellular domain of human TRAIL-receptor proteins immobilised on 96-well plates was characterised using an ELISA format. Specific binding of HGS-ETR1 to the TRAIL-R1 protein but not to other TRAIL receptors was observed (Figure 1A). The specificity of HGS-ETR1 binding and activity was also analysed in cell-based assays. Binding of HGS-ETR1 to TRAIL-R1 on the surface of SW480 tumour cells was detected by flow cytometry. HGS-ETR1 binding was specific for TRAIL-R1 as only soluble TRAIL-R1-Fc protein, but not other soluble TRAIL receptors, blocked binding (Figure 1B). Agonistic activity of HGS-ETR1 was shown in *in vitro* tumour cell viability assays where significant cell death occurred after a 48-hour treatment of SW480 cells with HGS-ETR1. No loss of viability was observed by treatment with an isotype control antibody. Addition of soluble TRAIL-R1, but not soluble TRAIL-R2, TRAIL-R3, TRAIL-R4 or OPG, blocked killing of SW480 cells by HGS-ETR1 (Figure 1C).

### HGS-ETR1 reduces viability and activates apoptotic signalling in human tumour cell lines

**Figure 2:**
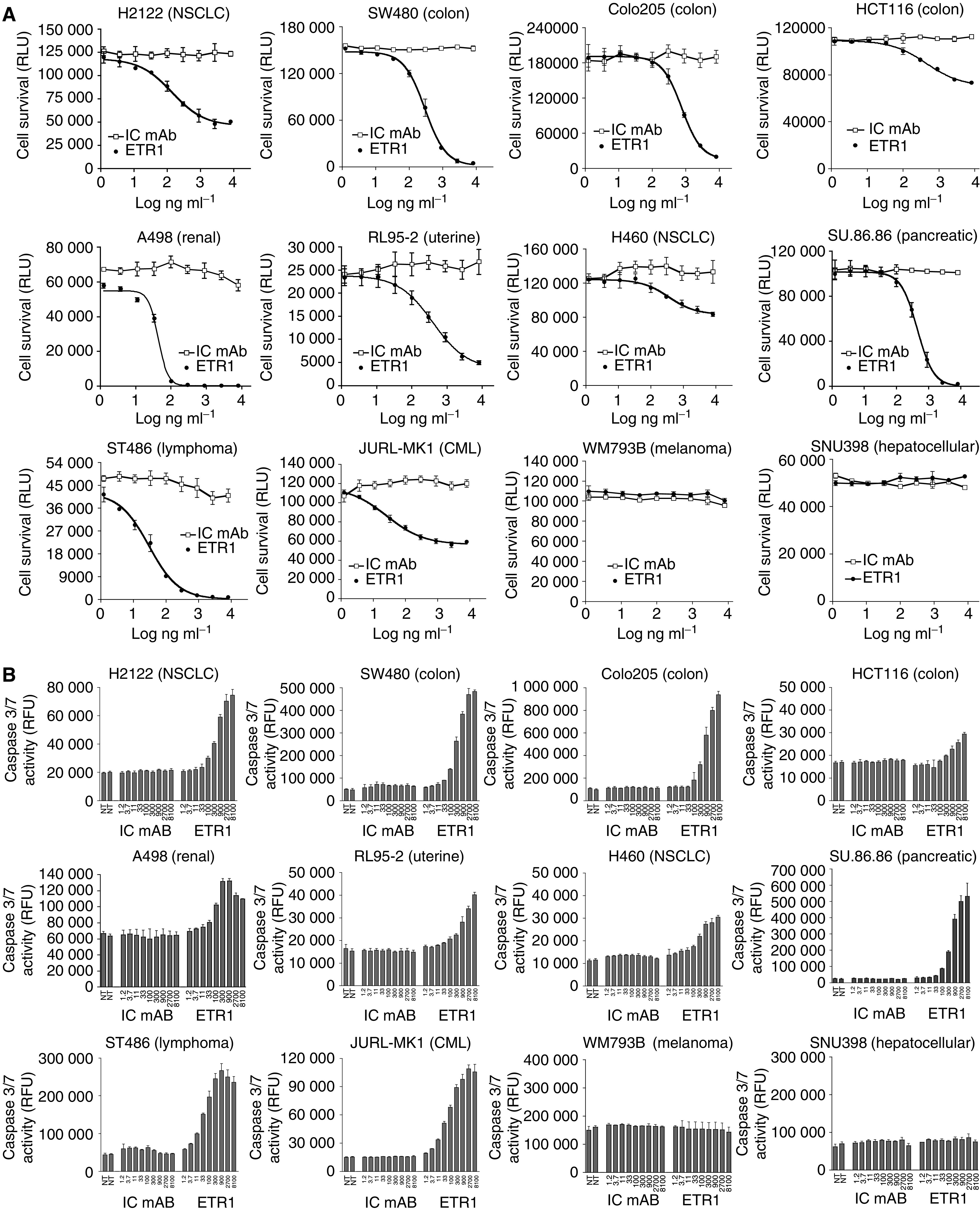

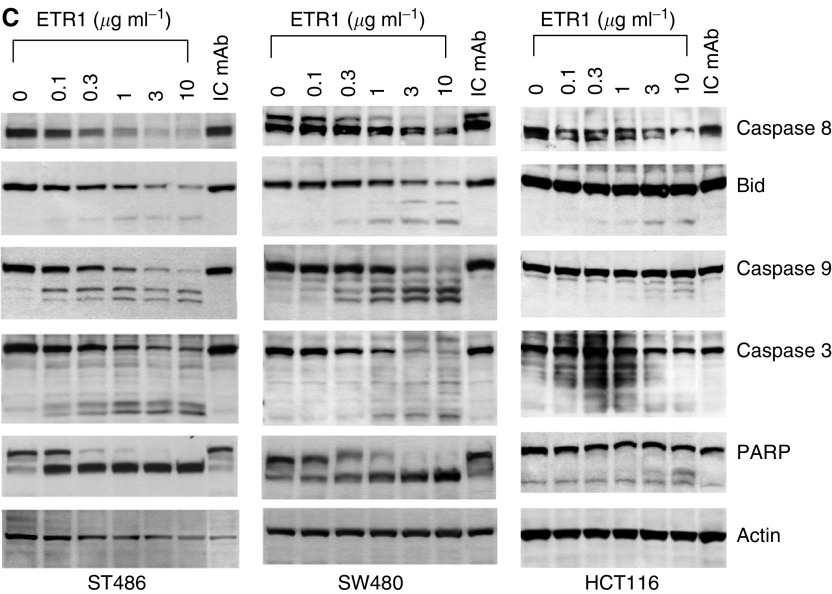
HGS-ETR1 induces tumour cell killing and apoptotic signalling. (**A**) SW480, Colo205, HCT116, H2122, H460, SU.86.86. RL95-2, ST486, JURL-MK1, A498, SNU398, and WM793B cells were plated in 96-well plates at approximately 1 × 10^4^ cells well^−1^, and cultured overnight. Indicated concentrations of HGS-ETR1 (closed circles), or isotype control antibody (IC mAb, open squares) were added and cell viability, measured as relative light units (RLU), was determined after 48 h. (**B**) Cells were treated as in viability assays (ng ml^−1^) and caspase 3/7 activity was measured as relative fluorescent units (RFU) after 6 h of treatment. The viability and caspase data are the mean±s.d. of triplicate samples from matching representative experiments. (**C**) ST486, SW480, and HCT116 cells were plated on 100-mm plates, cultured overnight and indicated concentrations of HGS-ETR1 or isotype control antibody (10 *μ*g ml^−1^) were added. After 4 h of treatment, cell lysates were prepared, normalised for protein concentration and Western analysis was performed. The actin protein levels are shown as a control for protein loading.

HGS-ETR1 induction of cell killing and mechanism of action were analysed in multiple tumour cell lines (SW480, Colo205, HCT116, H2122, H460, SU.86.86, RL95-2, ST486, JURL-MK1, A498, SNU398, and WM793), representing various tumour types (Table 1). All of the cell lines, with the exception of the WM793 melanoma cell line, expressed detectable levels of cell surface TRAIL-R1 by FACScan analysis (Table 1). Treatment with various concentrations of HGS-ETR1 resulted in a dose-dependent reduction of cell viability at 48 h (Figure 2A) preceded by activation of caspase 3/7 at 6 h (Figure 2B) in most cell lines. Sensitivity to HGS-ETR1 varied among these cells. For example, HGS-ETR1 treatment substantially decreased viability of ST486, and SW480, whereas H460 and HCT116 showed lesser sensitivity to HGS-ETR1-induced killing. HGS-ETR1 induced apoptotic signalling as measured by caspase 3/7 activation in all cell lines that were killed by HGS-ETR1 treatment. HGS-ETR1 did not induce caspase activity or reduce cell viability in WM793 or SNU-398 cells.

To more fully characterise the signalling pathways used by HGS-ETR1, activation of intracellular apoptotic signalling molecules was determined by Western analysis in three cell lines. Dose-dependent activation of caspases, as determined by a decrease in procaspase 8 levels and cleavage of caspase 9 and caspase 3, was observed after a 4-h treatment of ST486 and SW480 cell lines with HGS-ETR1 (Figure 2C). HGS-ETR1 treatment also resulted in cleavage of Bid, a substrate of caspase 8, and PARP, a substrate of caspase 3. The activation of these pathways by HGS-ETR1 was clearly evident in ST486 and SW480. In contrast, in HCT116 cells, minimal activation of death signalling proteins was detected at high concentrations of HGS-ETR1 (Figure 2C).

### HGS-ETR1 enhancement of chemotherapeutic agent activity *in vitro*

**Figure 3:**
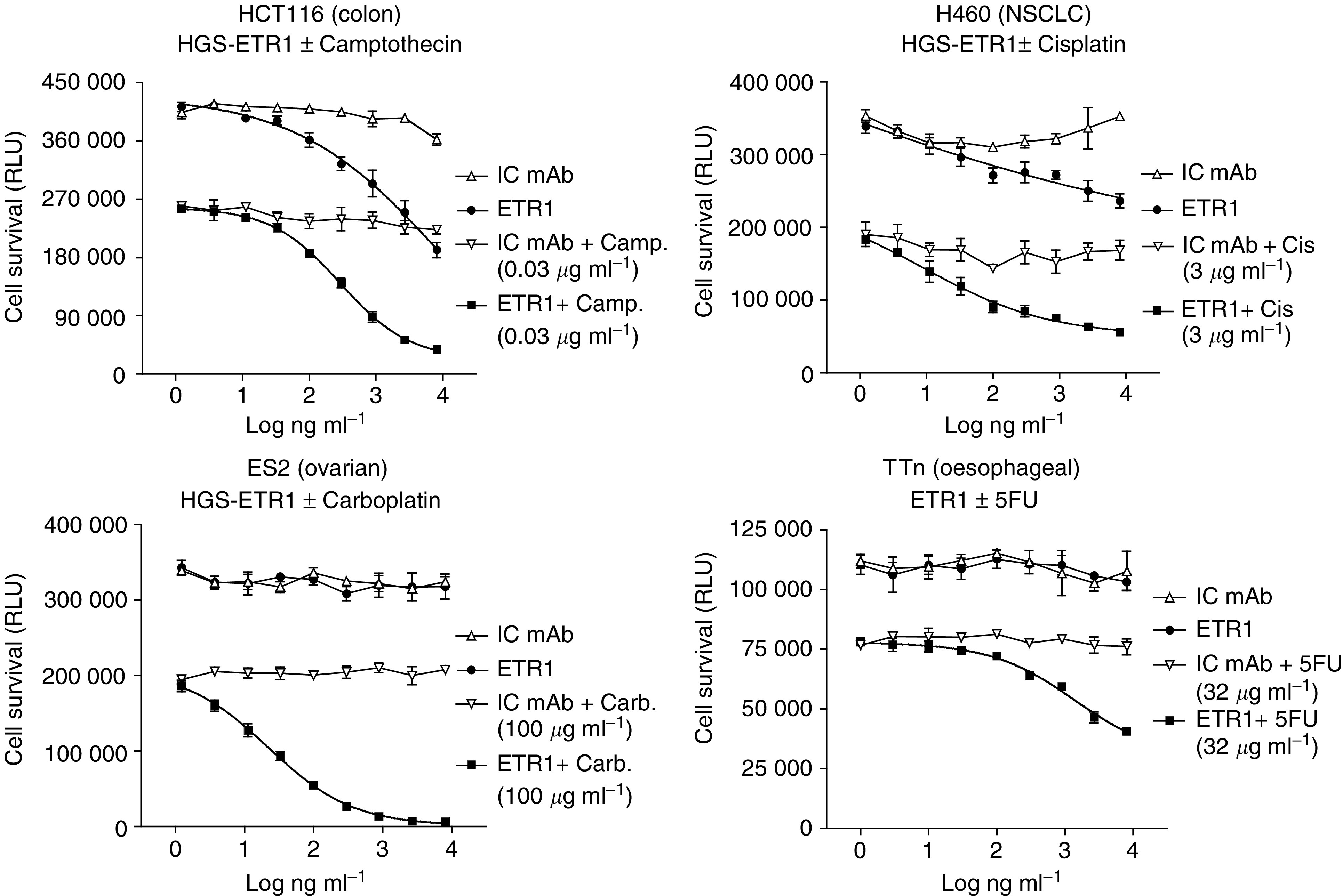
Combination of HGS-ETR1 with chemotherapeutic agents enhances tumour cell killing *in vitro*. HCT116, H460, ES2, and TTn cells were plated in 96-well plates at approximately 1 × 10^4^ cells well^−1^ and cultured overnight. Indicated concentrations of HGS-ETR1 (closed circles), isotype control antibody (IC mAb, open upward triangles); HGS-ETR1 plus indicated chemotherapeutic drug (closed squares), or IC mAb plus chemotherapeutic drug (open downward triangles) were added and cell viability, measured as relative light units (RLU), was determined after 48 h of treatment. Data are the mean±s.d. of triplicate samples.

Combination treatment of HGS-ETR1 with chemotherapeutic agents *in vitro* was analysed in HGS-ETR1-sensitive (HCT116 colon, H460 NSCLC) and -insensitive (ES2 ovarian, and TTn oesophageal) tumour cell lines. Cells were treated with various concentrations of control antibody or HGS-ETR1 either alone or in combination with camptothecin (HCT116), cisplatin (H460), carboplatin (ES2), or 5-fluorouracil (TTn) and the cell viability was determined after 48 h (Figure 3). Chemotherapeutic drug concentrations were used that had been previously established to give approximately 50% cell killing by drug alone (data not shown). In each case, the treatment with HGS-ETR1 plus chemotherapeutic drug resulted in enhanced cell killing, which was greater than the effect of either agent alone. Combination treatment substantially enhanced chemotherapeutic drug cytotoxicity even in two cell lines (TTn and ES2) where HGS-ETR1 treatment alone did not decrease cell viability, suggesting a synergistic interaction between HGS-ETR1 and the chemotherapeutic agents.

### HGS-ETR1 reduces the growth of human tumours in xenograft models

**Figure 4:**
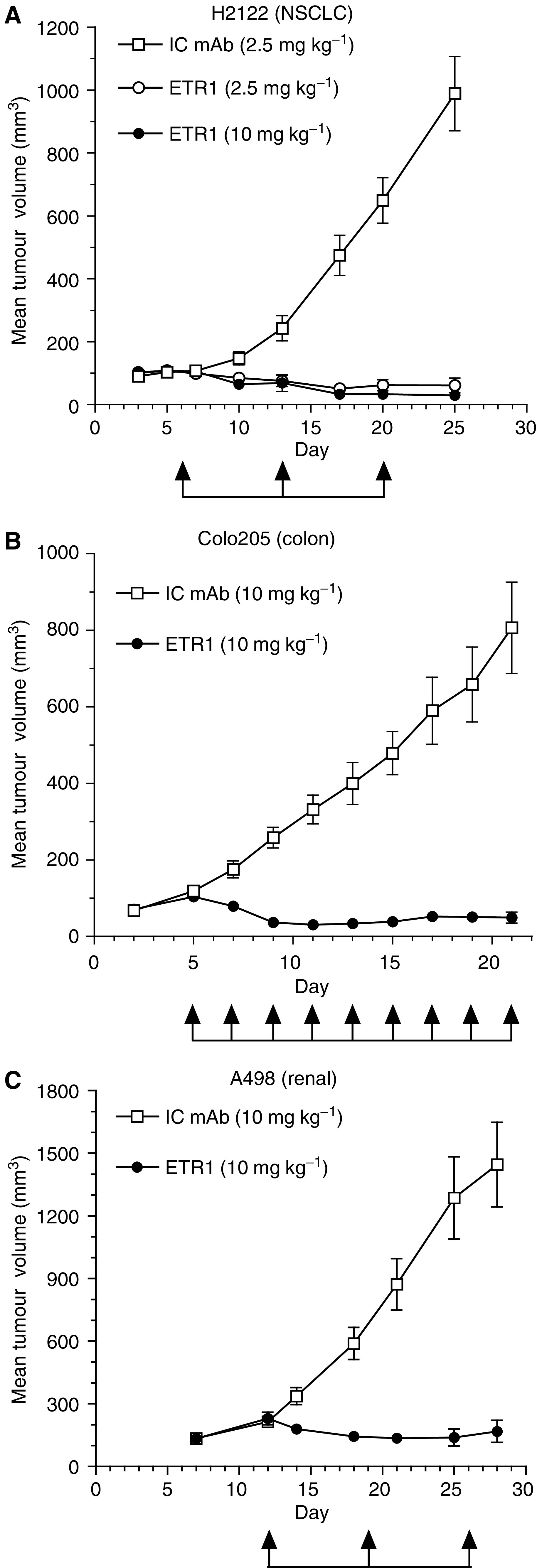
HGS-ETR1 represses the growth of lung, colon, and renal tumours *in vivo*. Athymic mice were injected s.c. at day 0 with H2122 (**A**), Colo205 (**B**), or A498 (**C**) tumour cells. After tumours had reached approximately 100 mm^3^, mice were administered isotype control monoclonal antibody (IC mAb, open squares) or HGS-ETR1 (circles) by i.v. injection at the indicated times (arrows) and doses. Eight to 10 mice were included in each group. Each time point represents the mean value (±s.e.m.) of the tumour sizes within the treatment group on the day of measurement.

HGS-ETR1 *in vivo* antitumour activity was evaluated in NSCLC (H2122), colon (Colo205), and renal (A498) tumours in xenograft models in athymic nude mice. Tumours were pre-established s.c. to a volume of approximately 100 mm^3^ before initiation of antibody treatments. Three weekly treatments of HGS-ETR1 at a concentration of 2.5 or 10 mg kg^−1^ showed strong single-agent antitumour activity in the H2122 NSCLC xenograft model, with significant tumour regression observed after a single injection of HGS-ETR1 (Figure 4, *P*<0.0001). Tumour volume was reduced approximately 50% by day 10, 4 days after the first injection of HGS-ETR1 and by day 25, there was a 97% reduction in tumour volume for the 10 mg kg^−1^ dose of HGS-ETR1. In addition, tumour regrowth was suppressed for several weeks after termination of HGS-ETR1 treatment (data not shown). HGS-ETR1 also induced tumour regression in colon and renal xenograft tumour models. Treatment with a 10 mg kg^−1^ dose of HGS-ETR1, every other day or once a week, respectively, dramatically reduced tumour volumes in Colo205 colon (*P*<0.0001 at day 21) and A498 renal (*P*<0.0001 at day 28) xenograft tumour models compared to the isotype control antibody (Figure 4).

**Figure 5:**
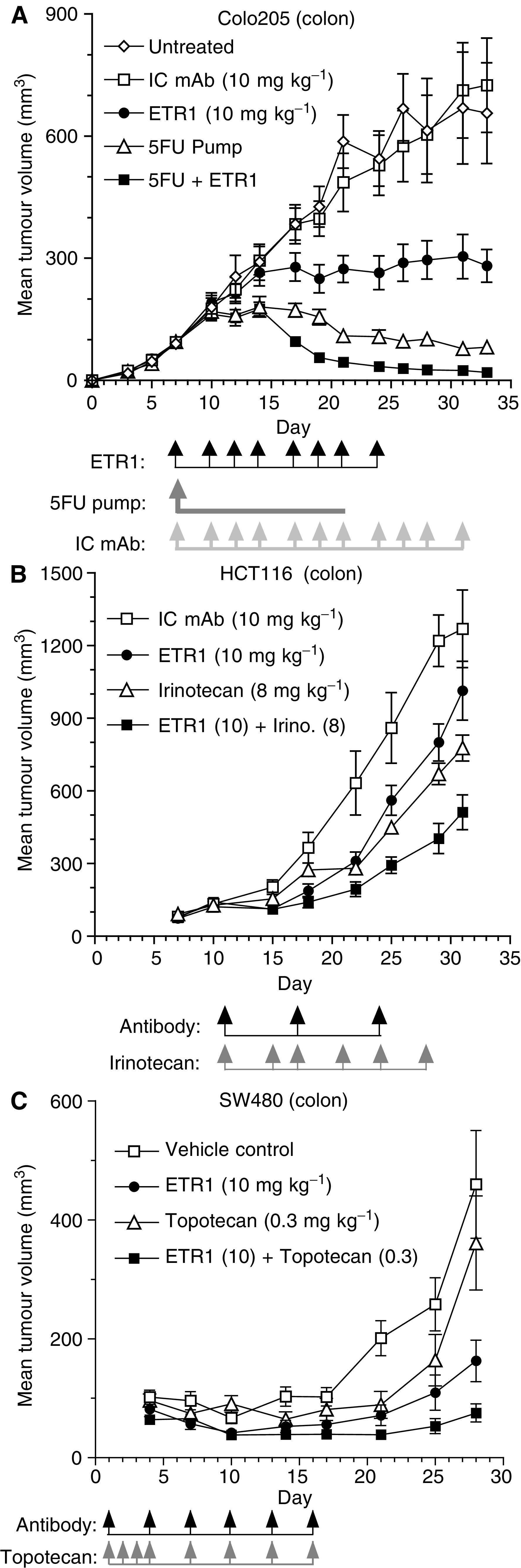
Enhanced antitumour effect by combination of chemotherapeutic agents and HGS-ETR1. Athymic mice were injected s.c. on day 0 with tumour cells. For the Colo205 and HCT116 models, tumours were allowed to grow to approximately 100 mm^3^ before treatment. Chemotherapeutic agents, isotype control monoclonal antibody (IC mAb), or HGS-ETR1 were administered at the indicated times (arrows) and concentrations. Eight to 10 mice were included in each group. For the SW480 *de novo* model, a loading dose of HGS-ETR1 (20 mg kg^−1^) was administered i.v. on day 1. Topotecan was administered i.p. at a dose of 0.3 mg kg^−1^ on days 1–3. Subsequently, HGS-ETR1 (10 mg kg^−1^) and topotecan (0.3 mg kg^−1^) were administered i.p. on days 4, 7, 10, 13, and 16. One group of control mice was injected only with vehicle control. Six mice were included in each group. For all models, each time point represents the mean value (±s.e.m.) of the tumour sizes within the treatment group on the day of measurement.

The ability of HGS-ETR1 to enhance the *in vivo* antitumour activity of three different chemotherapeutic agents was tested in three independent colon xenograft models (Figure 5). HGS-ETR1 in combination with 5FU was tested in the Colo205 xenograft model (Figure 5A). Tumours of approximately 100 mm^3^ were pre-established, and animals were treated with 5FU alone (continuous infusion mini-pump, 1.25 mg kg^−1^ h^−1^, days 7–21), HGS-ETR1 alone (i.v., 10 mg kg^−1^, days 14, 17, 19, 21, 24, 27, 29, and 31), or combination of 5FU and HGS-ETR1, and compared to no treatment or treatment with isotype control mAb (i.v., 10 mg kg^−1^, days 7–31). Tumour size in nontreated animals was not significantly different from isotype control mAb treated. Both 5FU alone (*P*<0.001) and HGS-ETR1 alone (*P*<0.001) had a significant cytostatic effect on tumour growth compared to control. For the HGS-ETR1+5FU combination treatment group, mice were pretreated with 5FU for 7 days before treatment with HGS-ETR1 and resulted in an immediate decrease in tumour volume evident at day 17. The overall effect on tumour volume after combining HGS-ETR1 and 5FU was significantly different when compared to either agent alone (*P*<0.001).

The effect of combination of HGS-ETR1 with irinotecan was examined in pre-existing HCT116 colon tumours (Figure 5B). Tumours of approximately 100 mm^3^ were established and animals were treated with irinotecan (i.p., 8 mg kg^−1^), HGS-ETR1 (i.v., 10 mg kg^−1^), or isotype control antibody (i.v., 10 mg kg^−1^). At day 31, treatment with irinotecan alone resulted in a 38.8% reduction in tumour growth compared to control antibody (*P*<0.0113). HGS-ETR1 treatment showed a 20.1% reduction in tumour volume, which was not significantly different from that of the control antibody group (*P*<0.2248). The combination of HGS-ETR1 plus irinotecan resulted in a significant reduction of tumour growth (59.7%) when compared with control antibody (*P*<0.0007), irinotecan alone (*P*<0.0103), or HGS-ETR1 alone (*P*<0.007).

The effect of HGS-ETR1 and topotecan alone or in combination was tested in a *de novo* SW480 colon tumour cell model (Figure 5C). On day 1, 24 h after SW480 tumour cell implantation, a loading dose of HGS-ETR1 (20 mg kg^−1^) was administered i.v. Topotecan (0.3 mg kg^−1^) was administered i.p. on days 1, 2, and 3. Topotecan and HGS-ETR1 (10 mg kg^−1^) alone or in combination were administered i.p. on days 4, 7, 10, 13, and 16. At day 28, when compared to vehicle control, topotecan alone did not show a significant effect (19.2% reduction in tumour volume; *P*<0.349), but HGS-ETR1 treatment alone (51.5% reduction; *P*<0.040), or coadministration of HGS-ETR1 and topotecan (70.7% reduction; *P*<0.007), significantly inhibited the growth of SW480 tumours. The reduction of tumour growth by the combination of HGS-ETR1 plus topotecan was significantly greater than topotecan alone (*P*<0.0194); however, significance was not reached when the combination was compared to HGS-ETR1 alone (*P*<0.0907).

### Pharmacokinetics of HGS-ETR1 in normal BALB/c mice following a single intravenous injection

**Figure 6:**
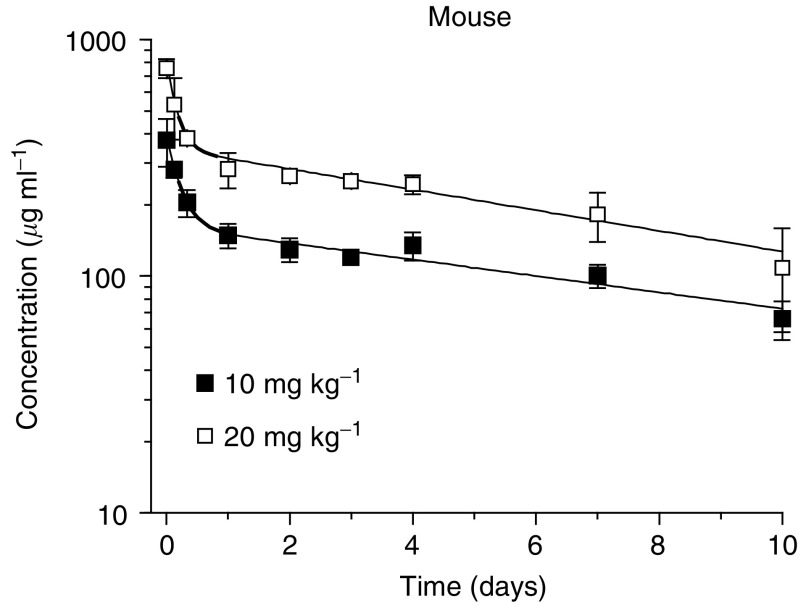
Pharmacokinetics of HGS-ETR1 in mice. Serum concentrations of HGS-ETR1 were determined in BALB/c mice following i.v. injection of 10 or 20 mg kg^−1^. Male BALB/c mice were injected i.v. with either 10 or 20 mg kg^−1^ HGS-ETR1, and serum concentrations were followed for 10 days. Data points are the mean (±s.d.) and solid lines represent the fit of a two-compartment pharmacokinetic model to the data.

The pharmacokinetics of HGS-ETR1 was evaluated in Balb/c mice following a single i.v. injection of 10 or 20 mg kg^−1^. Serum concentrations of HGS-ETR1 were determined by ELISA (Figure 6). The solid line shows a fit of a two-compartment pharmacokinetic model to the serum concentration data. Pharmacokinetic parameters are listed in Table 2. The beta, or elimination, phase contributes approximately 98% of the total area under the curve. The terminal half-life (*t*_1/2, *β*_) of HGS-ETR1 in mice is 6.9–8.7 days, the clearance is 4.81–5.66 ml day^−1^ kg^−1^, and the steady-state volume of distribution (*V*_ss_) is 55.3–59.0 ml kg^−1^. The *C*_max_ and AUC are approximately proportional to dose. The *t*_1/2, *β*_, CL, *V*_i_, and *V*_ss_ are not significantly different between dose groups, indicating that the pharmacokinetics of HGS-ETR1 were linear over the limited range of doses tested.

## DISCUSSION

Engagement of cell surface death receptors by TRAIL is the initial step in the induction of intracellular death signalling pathways and apoptosis (Srivastava, 2001; Ozoren and El-Deiry, 2003). Mouse agonistic mAbs generated against human TRAIL-R1 and TRAIL-R2 also induce apoptosis in human tumour cells (Ichikawa *et al*, 2001; Ohtsuka *et al*, 2003; Yagita *et al*, 2004). In this report, we examine the activity of HGS-ETR1, a fully human TRAIL-R1 agonistic mAb, and our results confirm and extend these previous observations. HGS-ETR1 bound specifically to TRAIL-R1 on the surface of human tumour cells and induced apoptosis of diverse tumour types. The WM793B melanoma cell line lacked measurable cell surface TRAIL-R1 expression and did not show sensitivity to HGS-ETR1. Despite the very high levels of TRAIL-R2 expression on this cell line, HGS-ETR1 was unable to affect WM793B cell viability. This data and the specific binding of HGS-ETR1 to TRAIL-R1 support the theory that HGS-ETR1 activity is mediated only through TRAIL-R1. In the cell lines in which HGS-ETR1 induced cell death, HGS-ETR1 also triggered caspase activation, indicating that induction of apoptosis is the primary mechanism through which HGS-ETR1 kills tumour cells. HGS-ETR1 induced activation of both the extrinsic pathway, as demonstrated by caspase 8 activation, and the intrinsic pathway as evidenced by caspase 9 activation, showing that specific activation of the TRAIL-R1 death receptor can lead to signalling though both apoptotic pathways. The activation of these cell death cascades led to measurable and concomitant activation of the executioner or terminal caspase 3. The use of a TRAIL-R1-specific high-affinity mAb demonstrated that TRAIL-R1 can mediate a dominant and potent activation of apoptosis, contrary to recent data suggesting that there is a preferential activation of TRAIL-R2 for the induction of apoptosis (Kelley *et al*, 2005).

Although TRAIL-R1 cell surface expression was required for the cytotoxic activity of HGS-ETR1, TRAIL-R1 expression levels did not predict the level of sensitivity. In general, a higher expression of TRAIL-R1 was seen in the most sensitive cell types; however, we found that there are cell lines in which there is a divergence between receptor level and HGS-ETR1 activity. For example, some cells with lower levels of TRAIL-R1 (RL95-1) were eliminated to a greater extent than cells with higher TRAIL-R1 expression (H460 or TTn). Additionally, one cell line (SNU398) with strong TRAIL-R1 cell surface expression staining was completely resistant to HGS-ETR1. Similar divergent responses in cell viability and cell surface TRAIL death receptor levels were demonstrated for TRAIL (Keane *et al*, 1999; Srivastava, 2001; Jang *et al*, 2003; Song *et al*, 2003), and variations in sensitivity of glioma cell lines to an anti-TRAIL-R2 mAb were not correlated with TRAIL-R2 protein expression (Kaliberov *et al*, 2004). The reason for this spectrum of response to TRAIL-R activation is due to the complex downstream regulation of the cell death pathway. Intracellular levels of pro- and antiapoptotic factors such as cFLIP (Kim *et al*, 2000; Sayers *et al*, 2003), XIAP (Zhang *et al*, 2001; Chawla-Sarkar *et al*, 2002), BCL-2 family members (Burns and El-Deiry, 2001; LeBlanc *et al*, 2002), and DISC formation and caspase 8 activity (Trauzold *et al*, 2003) have been reported to regulate cell sensitivity to TRAIL-induced programmed cell death in both tumour and normal cells. Although TRAIL decoy receptors have been implicated in the regulation of TRAIL signalling, the presence of decoy receptors (DcR1, DcR2) are not a factor in resistance to HGS-ETR1 activity, as HGS-ETR1 does not bind to either of these receptors (Figure 1). The levels and activity of pro- and antiapoptotic intracellular factors, in addition to the levels of TRAIL-R1 receptor, will influence the ability of HGS-ETR1 to induce apoptosis in a given cell line. Further work will be needed to identify the key factors that contribute to tumour cell sensitivity to HGS-ETR1.

*In vivo*, HGS-ETR1 had potent antitumour activity in pre-established colon, lung, and kidney xenograft cancer models. In these experiments, HGS-ETR1 was administered at 2- or 7-day intervals; given the half-life of HGS-ETR1 in mice (∼5–7 days), we would expect HGS-ETR1 to accumulate using these schedules. The tumour regression observed in these models demonstrated that these treatments were pharmacodynamically effective. HGS-ETR1 was highly active in the H2122 NSCLC model and induced a rapid decrease in tumour volume after a single injection of HGS-ETR1 (2.5 or 10 mg kg^−1^), eliciting significant tumour regression. Tumour regrowth was repressed for 20 days after the last HGS-ETR1 treatment, suggesting that the effects of HGS-ETR1 on tumour cell growth can extend beyond the treatment phase. The lack of tumour regrowth could be attributed to several factors including activity of the residual HGS-ETR1, and the significant loss of tumour cell number, tumour burden, and tumour vasculature during the initial tumour regression. HGS-ETR1 alone, at a concentration of 10 mg kg^−1^, also dramatically reduced tumour volume in the Colo205 colon and A498 renal models demonstrating that HGS-ETR1 had strong antitumour activity in multiple, diverse tumour types.

As cancer therapy in the clinic often involves the use of multiple therapeutic regimens, it was of interest to determine if HGS-ETR1 in combination with commonly used anticancer agents could enhance HGS-ETR1 activity *in vitro* and *in vivo*. Previous studies have shown that chemotherapeutic agents enhance TRAIL-induced cell killing of various tumour cells *in vitro* and *in vivo* (Naka *et al*, 2002; Arizono *et al*, 2003; Ray and Almasan, 2003; Singh *et al*, 2003). Combination treatment with chemotherapeutic agents *in vitro* also enhance murine TRAIL-R1 and TRAIL-R2 agonistic antibody-induced cell killing (Ohtsuka *et al*, 2003), and combination of a murine TRAIL-R2 antibody with adriamycin or paclitaxel showed greater inhibition of tumour growth compared to antibody alone in a breast cancer model *in vivo* (Buchsbaum *et al*, 2003).

Combination of HGS-ETR1 with different chemotherapeutic agents in *in vitro* assays using HCT116 and H460 cells resulted in enhanced cell killing compared to HGS-ETR1 or drug alone. In two cell lines (ES2 and TTn), substantial enhancement of carboplatin- or 5FU-induced cytotoxicity was observed after cotreatment with HGS-ETR1, even in the absence of detectable activity of HGS-ETR1 alone, demonstrating a synergistic interaction between HGS-ETR1 and these chemotherapeutic agents. *In vivo*, enhanced efficacy of combination treatments of HGS-ETR1 with three different chemotherapy agents was demonstrated in three separate colon xenograft tumour models. In the Colo205 model, 5FU was given for 7 days via infusion, prior to HGS-ETR1 treatment to emulate a current therapeutic delivery mode for 5FU in colon cancer. Combination of HGS-ETR1 treatment with 5FU pretreatment was effective in inducing a pronounced tumour regression, suggesting that combination of chemotherapy and HGS-ETR1 do not have to be given concurrently for efficacy. Interestingly, this regression occurred immediately after injection of HGS-ETR1, suggesting that the antitumour action of the mAb to induce apoptosis and reduce tumour volume is rapid. Various mechanisms have been proposed for the enhanced cell death observed by combination of chemotherapy with TRAIL, including upregulation of TRAIL-R1 and TRAIL-R2 (Bouralexis *et al*, 2001, 2003; Nimmanapalli *et al*, 2001; Singh *et al*, 2003), increased levels of the proapoptotic protein, Bak (LeBlanc *et al*, 2002) and suppression of prosurvival pathways (Asakuma *et al*, 2003). We have initiated experiments to investigate the mechanisms responsible for the enhancement of antitumour activity by HGS-ETR1 in combination with chemotherapeutic agents.

The pharmacokinetic profile of HGS-ETR1 indicated that the half-life of HGS-ETR1 was 6.9–8.7 days. For comparison, the reported half-life of the TRAIL ligand that binds both TRAIL-R1 and TRAIL-R2 is 3.6 min in mice (Kelley *et al*, 2001). The terminal half-life of the HGS-ETR1 fully human monoclonal antibody is similar to the 6–8-day half-life in mice reported for endogenous IgG2a (Viera and Rajewsky, 1988) and the 6-day half-life in nude mice reported for humanised IgG1 monoclonal antibodies against VEGF or TAG-72 (Kashmiri *et al*, 1995; Lin *et al*, 1999). HGS-ETR1 showed a clearance of 4.81–5.66 ml day^−1^ kg^−1^, and the *V*_ss_ was approximately 60 ml kg^−1^. This indicates that HGS-ETR1 does distribute to tissues, as the *V*_ss_ represents a space greater than plasma volume (40 ml kg^−1^), although it is restricted to a space less than the total extracellular fluid volume of 170 ml kg^−1^ (Levine, 1990).

Overall, we have shown that HGS-ETR1, the first reported fully human agonistic TRAIL-R1 mAb, specifically and exclusively bound to TRAIL-R1, and potently induced apoptosis *in vitro* in a broad range of human tumour cell types via activation of both intrinsic and extrinsic signalling cascades. HGS-ETR1 had an effective pharmacokinetic half-life, and induced rapid tumour regression in multiple xenograft tumour models alone and in combination with chemotherapeutic agents. These data indicate that HGS-ETR1 has significant potential as a cancer therapeutic agent. HGS-ETR1 is currently being evaluated in Phase I/II clinical trials in patients with advanced solid or haematological tumours.

## Figures and Tables

**Table 1:** TRAIL-R1 and TRAIL-R2 cell surface expression on tumour cell lines

**Cell line**	**Tumour type**	**TRAIL-R1 expression**	**TRAIL-R2 expression**
SW480	Colon adenocarcinoma	3.4±1.3	5.9±2.0
H2122	Non-small-cell lung carcinoma	3.1±0.2	7.1±0.3
A498	Renal cell carcinoma	2.7±1.2	6.4±1.1
ST486	Burkitt's lymphoma	1.9±0.1	0.6±0.2
Colo205	Colon adenocarcinoma	1.8±0.6	7.4±2.1
SU.86.86	Pancreatic ductal carcinoma	1.8±0.4	2.9±0.2
H460	Non-small-cell lung carcinoma	1.7±0.5	4.0±1.0
SNU-398	Hepatocellular carcinoma	1.6±0.2	3.2±0.0
TTn	Oesophageal squamous carcinoma	1.45±0.4	1.0±0.3
HCT116	Colon adenocarcinoma	1.4±0.2	3.4±0.9
JURL-MK1	Chronic myeloid leukaemia	1.15±0.1	2.6±0.5
RL95-2	Uterine endometrial carcinoma	1.2±0.2	1.9±0.2
ES2	Ovarian clear cell carcinoma	1.1±0.1	4.9±1.7
WM793B	Melanoma	1.0±0.09	10.2±0.5

TRAIL-R1 and TRAIL-R2 expression was determined by FACs analysis and is expressed as the fold-increase in mean fluorescent signal of commercial mouse TRAIL-R1 or R2 antibody *vs* isotype control antibody. The data is the average±s.d. from two or more experiments.

**Table 2:** Pharmacokinetic parameters following IV injection of HGS-ETR1 in BALB/c mice (mean±s.e.m.)

**Parameter**	**10 mg kg^−1^**	**20 mg kg^−1^**	***P*-value**
AUC (day·*μ*g ml^−1^)	2080±175	3531±287	N/A
AUC per dose (day·kg ml^−1^)	0.280±0.018	0.177±0.014	0.1702
% AUC_*β*_ (%)	97.6	97.9	N/C
CL (ml day^−1^kg^−1^)	4.81±0.41	5.66±0.46	0.1726
*C*_max_ (*μ*g ml^−1^)	392±27	819±77	N/A
*C*_max_/dose (kg ml^−1^)	0.039±0.003	0.041±0.004	0.7124
*t*_1/2, *α*_ (days)	0.148±0.033	0.107±0.033	0.3829
*t*_1/2, *β*_ (days)	8.71±1.1	6.90±0.89	0.2054
MRT (days)	12.3±1.5	9.76±1.2	0.1908
*V*_i_ (ml kg^−1^)	25.5±1.8	24.4±2.4	0.7077
*V*_ss_ (ml kg^−1^)	59.0±3.1	55.3±3.6	0.4390

AUC=area under the curve; % AUC*_β_*=percent of AUC in beta phase; CL=clearance; *C*_max_=maximum concentration; *t*_1/2, *α*,=_half-life of the alpha phase; *t*_1/2, *β*_=half-life of the beta phase; MRT=mean residence time; *V*_i_=initial volume of distribution; *V*_ss_=steady-state volume of distribution; N/C=not calculated; N/A=not available.
